# Iron metabolism disorder and multiple sclerosis: a comprehensive analysis

**DOI:** 10.3389/fimmu.2024.1376838

**Published:** 2024-03-21

**Authors:** Chao Tang, Jiaxin Yang, Chaomin Zhu, Yaqi Ding, Sushuang Yang, Bingyang Xu, Dian He

**Affiliations:** ^1^ Department of Neurology, Affiliated Hospital of Guizhou Medical University, Guiyang, Guizhou, China; ^2^ School of Clinical Medicine, Guizhou Medical University, Guiyang, Guizhou, China

**Keywords:** multiple sclerosis, iron metabolism, Mendelian randomization, bioinformatic analysis, causal relationship

## Abstract

**Background:**

Multiple sclerosis (MS) is the most common chronic inflammatory disease of the central nervous system. Currently, the pathological mechanisms of MS are not fully understood, but research has suggested that iron metabolism disorder may be associated with the onset and clinical manifestations of MS.

**Methods and materials:**

The study utilized publicly available databases and bioinformatics techniques for gene expression data analysis, including differential expression analysis, weighted correlation network analysis, gene enrichment analysis, and construction of logistic regression models. Subsequently, Mendelian randomization was used to assess the causal relationship between different iron metabolism markers and MS.

**Results:**

This study identified IREB2, LAMP2, ISCU, ATP6V1G1, ATP13A2, and SKP1 as genes associated with multiple sclerosis (MS) and iron metabolism, establishing their multi-gene diagnostic value for MS with an AUC of 0.83. Additionally, Mendelian randomization analysis revealed a potential causal relationship between transferrin saturation and MS (p=2.22E-02; OR 95%CI=0.86 (0.75, 0.98)), as well as serum transferrin and MS (p=2.18E-04; OR 95%CI=1.22 (1.10, 1.36)).

**Conclusion:**

This study comprehensively explored the relationship between iron metabolism and MS through integrated bioinformatics analysis and Mendelian randomization methods. The findings provide important insights for further research into the role of iron metabolism disorder in the pathogenesis of MS and offer crucial theoretical support for the treatment of MS.

## 1 Introduction

Multiple sclerosis (MS) is the most common chronic inflammatory, demyelinating and neurodegenerative disease of the central nervous system(CNS), with a global prevalence exceeding 2 million, including approximately 400,000 cases in the United States (1). The pathological hallmark of this disease is the occurrence of disseminated “plaques of sclerosis” in multiple regions of the CNS, including white matter, gray matter, brainstem, spinal cord, and optic nerve (2, 3). The pathogenesis of MS is a complex and dynamic process among the immune system, glial cells, and neurons (4). These interactions lead to the pathophysiological changes in MS, including inflammatory demyelination, neuronal injury, and brain lesions (5). However, current treatments for MS mostly focus on controlling the degree of disease inflammation, with limited effectiveness in targeting inflammation (6).

Iron is an essential trace metal involved in the metabolism of catecholamine neurotransmitters and the formation of myelin in the nervous system (7). Multiple studies have indicated a link between brain iron deposition and normal aging as well as MS (8–10). MRI studies have shown excessive iron deposition in the gray matter of MS patients, which primarily concentrated in the basal ganglia. Additionally, iron deposition is also present in the white matter, particularly near lesion areas. Such iron deposition may be related to the development and clinical manifestations of the disease (11). The mechanism may involve the abnormal deposition of iron leading to oxidative stress, which further damage brain cells (12). Oxidative stress has been considered as part of the pathogenesis of MS, potentially contributing to demyelination and cell death. Therefore, abnormal iron deposition may be an important factor in the pathological process of MS (13). However, other studies have found that iron deficiency can also impact the pathology of MS. In a study investigating changes in iron content in the brains of MS patients, it was observed that the iron content in the deep gray matter showed a decreasing trend. Furthermore, the research revealed that as the disease progressed, the iron levels in patients with progressive MS were lower than those with relapsing-remitting MS (14). Similar results have been reported in other studies (15, 16). Systemic iron deficiency can also lead to MS. Multiple studies have indicated that the serum iron levels of MS patients are similar or lower compared to healthy control groups. This suggests that in MS patients, there may be a trend of decreased serum iron levels. The reduction in serum iron levels may be associated with the pathophysiological processes of MS (17). Iron deficiency in MS may involve variations in multiple genes, including TMPRSS6, HFE, TF, Dual Oxidase 2, CUBN, and SLC25A37. These genes encode proteins related to iron absorption, transport, and loss, which may result in iron deficiency (18, 19). Iron deficiency may further lead to MS because the majority of brain iron is found in oligodendrocytes. Therefore, iron deficiency may impact the pathophysiological processes in MS patients, including affecting the health and regeneration of oligodendrocytes and myelin sheaths (20). Additionally, Iron deficiency may lead to heightened oxidative metabolism activity in oligodendrocytes, as well as impact the synthesis and maintenance of enzymes that involve in oxidative metabolism and myelin sheath production (21).

In a word, iron metabolism dysfunction seems to be an important pathogenic mechanism in MS. We use comprehensive bioinformatic analysis and mendelian randomization (MR) methods to explore the relationship between iron and MS, with an aim to provide new evidence for further research on the pathogenic mechanism of iron in MS.

## 2 Materials and methods

### 2.1 Study design

First, we utilized a large volume of publicly available databases and conducted Differential Expression Analysis, Weighted Gene Correlation Network Analysis (WGCNA), Gene Ontology (GO) analysis, and Logistic Regression Model to demonstrate the role of iron in MS. Subsequently, we employed two-sample MR to assess the causal relationship between different iron metabolism indicators and MS. MR uses genetic variation as a proxy for risk factors. Therefore, effective instrumental variables (IVs) must satisfy three key assumptions in causal inference: (1) genetic variation is directly associated with the exposure; (2) genetic variation is unrelated to potential confounding factors between the exposure and the outcome; (3) genetic variation does not affect the outcome through pathways other than the exposure (22, 23).

**Figure 1:**
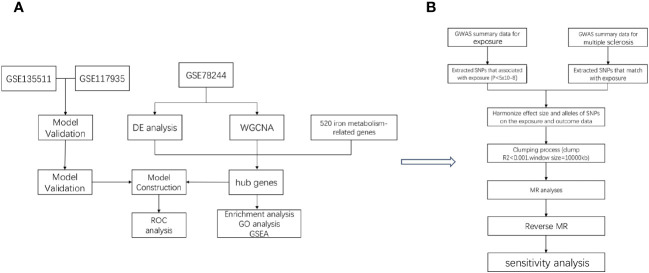
The workflow of the analyses. First, we conducted comprehensive informatics analysis **(A)**, and then we validated using Mendelian randomization method **(B)**.

In the study design, we used comprehensive informatics analysis and various MR methods to demonstrate the role of iron in MS. The study workflow is illustrated in **Figure 1**.

### 2.2 Bioinformatic analysis

#### 2.2.1 Data source

The gene expression data for MS was obtained from the NCBI Gene Expression Omnibus public database (GEO), specifically the study data GSE78244 (24), which analyzed the gene expression profiles of resting and activated CD4+ T cells from MS patients, including 28 MS patients and 28 healthy individuals. Data from GSE135511 and GSE117935 (25, 26) were also utilized for further validation. The iron-related data was derived from a study on pivotal genes and diagnostic models related to iron metabolism in Alzheimer’s disease, encompassing approximately 520 iron metabolism-related genes (27, 28).

#### 2.2.2 Differential expression analysis

Differential expression analysis of MS and control group samples was performed using the “limma” package in R software. Genes with p_adj < 0.05 and |logFC| > 0.585 were considered as differentially expressed genes (DEGs). Heatmaps and volcano plots of the DEGs were created using the “pheatmap” and “ggplot2” packages.

#### 2.2.3 Weighted correlation network analysis

The “WGCNA” package in R software was utilized to construct a gene co-expression network and identify relevant differentially expressed genes. WGCNA is a systems biology approach used to identify patterns in gene co-expression networks and group related genes into modules. This method, through the analysis of gene expression data, can reveal correlations between genes, aiding in the identification of gene modules associated with specific biological states. WGCNA is often used to discover biomarkers associated with diseases and to understand gene regulatory networks and signaling pathways (29).

#### 2.2.4 Gene enrichment analysis

To identify key genes associated with iron metabolism and MS, we used the “VennDiagram” package in R to find the overlap of Differentially Expressed Genes (DEGs), genes from WGCNA, and the iron metabolism gene set. We visualized the expression differences of these central genes between MS and control samples using violin plots, and employed t-tests or Mann-Whitney U tests to determine significance (p < 0.05). Subsequently, we conducted enrichment analysis of these hub genes to explore their impact on MS. We analyzed the involvement of these genes in biological processes (BP) using GO and presented the results using chord diagrams with the “GOplot” package in R. GSEA was also performed to unveil the specific functions of each gene, and the results were visualized using the “enrichplot” package in R. These analyses were carried out using the “clusterProfiler” package in R, with a filtering criterion of p_adj < 0.05.

#### 2.2.5 Logistic regression model

The logistic regression model is commonly used for automated disease diagnosis. In this study, we employed a logistic regression model with two response variables, where a response variable of 1 represented MS samples and 0 represented control samples. We initially conducted stepwise regression analysis to simplify the model by eliminating non-significant factors and retaining only significant factors. Stepwise regression iteratively added or removed variables from the model to minimize the Akaike information criterion (AIC). Subsequently, logistic regression was used to establish the relationship between these significant factors and the response variable. Finally, we evaluated the diagnostic performance of the model using the “stats” and “pROC” packages in R software, by plotting the receiver operating characteristic (ROC) curve and calculating the area under the ROC curve to assess the model’s performance.

### 2.3 MR study

#### 2.3.1GWAS data source

The exposures included ferritin, iron, transferrin saturation, and transferrin. There exposures were derived from a study on the impact of new loci affecting iron homeostasis on disease in a European population, which incorporated data from 11 cohorts comprising 23,986 individuals (30). Our study included 2,036,124 SNPs from this research. The targeted outcomes in this study stemed from an investigation of a novel MS susceptibility locus. The study encompassed a total of 4888 cases and 10,395 controls, with GWAS summary data containing 7,910,365 SNPs (31). Our validation data were obtained from the International MS Genetics Consortium’s study on risk allele genes for MS which identified their GWAS summary data containing 327,095 SNPs through whole-genome research (32).

#### 2.3.2 IV selection

We established criteria for IV selection to ensure the accuracy and effectiveness of the causal relationship between exposure and outcome. Firstly, we set the genome-wide significance threshold at a p-value of <5e-06, included only SNPs meeting this criterion as exposure and outcome IVs in the MR study. Secondly, we used the TwoSampleMR R package with r² = 0.01 and kb = 10000 to ensure the independence of the selected IVs and reduce linkage disequilibrium effects from random allele distribution. Additionally, considering the magnitude and precision of the SNP's genetic effect on the trait, we calculated the F-statistic using the formula F = R^2^(N − 2)/(1 − R^2^) to assess the strength of each SNP, through which we could estimate the proportion of SNP-explained trait variance. We excluded SNPs with an F-statistic less than 10, as only an F-statistic greater than 10 indicates sufficient strength to ensure the validity of the SNP.

#### 2.3.3 MR methods

MR is a method that utilizes genetic instruments to study causal relationships between modifiable exposures and outcomes. The analysis in our study employed five MR methods, including the ratio test, Inverse Variance Weighted (IVW) method, weighted generalized linear regression, weighted median method, and Mendelian Randomization Egger regression. Among these, the IVW method is the most crucial, as it calculates the inverse variance-weighted average of ratio estimates from multiple SNPs. The IVW method assumes that all SNPs are valid instruments, or any bias between instruments is balanced. It can provide estimates using fixed-effects or random-effects models. By employing these five MR methods, we aimed to minimize bias as much as possible and obtain reliable estimates of the causal relationship between the exposure of interest and the outcome.

### 2.4 Statistical methods

To obtain reliable causal relationships, we conducted a meta-analysis of the different results obtained from the initial and replication stages of the MR analysis (33). In this meta-analysis, we initially used a fixed-effects model and only employed a random-effects model in the presence of heterogeneity, with a statistical significance cutoff of p < 0.05 indicating meaningful results.

For sensitivity analysis, we measured heterogeneity using the Cochran Q method. In cases of significant heterogeneity (p < 0.05), we conducted MR analysis using a random-effects model; otherwise, we used a fixed-effects model. MR-Egger regression was employed to assess potential pleiotropy of the SNPs used as IVs. In MR-Egger regression, when the intercept term p < 0.05, it indicates the presence of directional pleiotropy. We also used the MR-PRESSO test to assess horizontal pleiotropy, with p < 0.05 indicating its presence. We used Radial MR to test for outliers, which can more directly detect and visualize outliers. Finally, for the robustness of the results, we conducted a leave-one-out analysis. This analysis included removing each SNP one at a time and then conducting MR analysis to determine if individual SNPs significantly affected the results. To explore whether MS is causally related to established iron levels, we also conducted reverse MR analysis (using MS as the exposure and established iron levels as the outcome).

In the comprehensive informatics analysis, t-tests and Mann-Whitney U tests were chosen based on the normality of the data distribution. Typically, the significance level is defined as p < 0.05.

All statistical analyses in this study were conducted using the R software package (v4.2.1) within the R programming language.

## 3 Results

### 3.1 Acquisition of hub genes related to iron metabolism in MS

**Figure 2:**
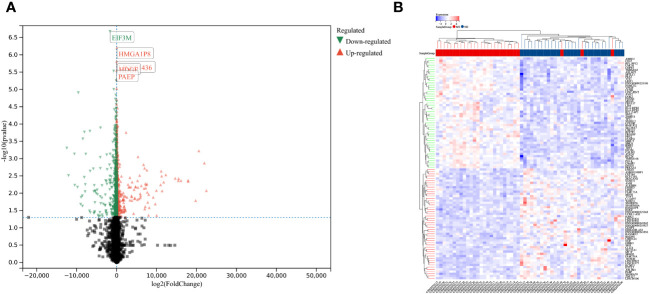
Differentially expressed genes between MS and control group samples. **(A)** Red genes represent significantly high expression in MS, green genes represent significantly high expression in the control group, and gray genes indicate insignificant changes. **(B)** The heatmap shows the top 50 genes significantly highly expressed in MS or control group samples.

**Figure 3:**
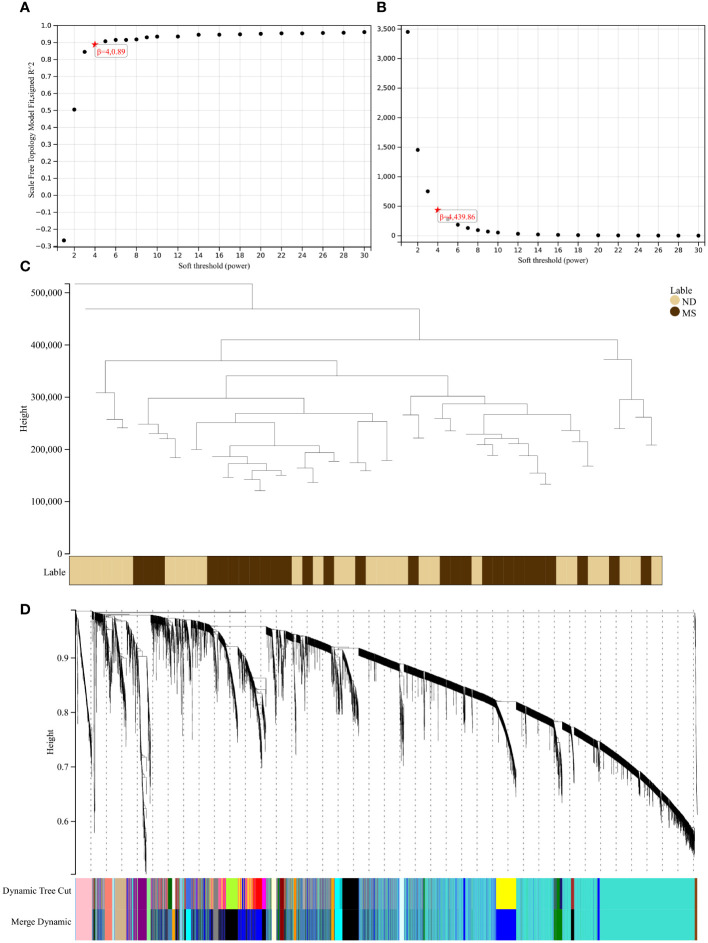
Results of the WGCNA. **(A)** The corresponding scale-free topological model fit indices at different soft threshold powers. **(B)** The corresponding mean connectivity values at different soft threshold powers. **(C)** Cluster dendrogram of samples. **(D)** Cluster dendrogram of genes.

**Figure 4:**
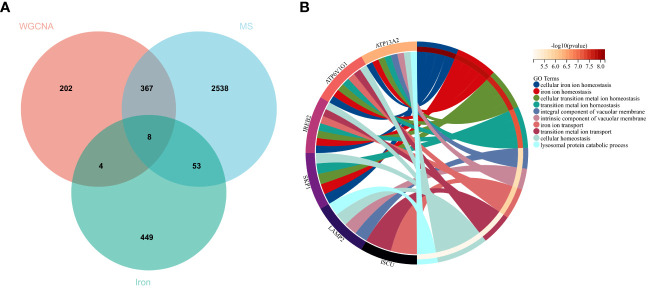
Hub genes and GO analysis. **(A)** Eight hub genes were obtained by taking the intersections of the DEGs, WGCNA, and iron metabolism-related genes. **(B)** Biological processes in which the hub genes were involved.

**Figure 5:**
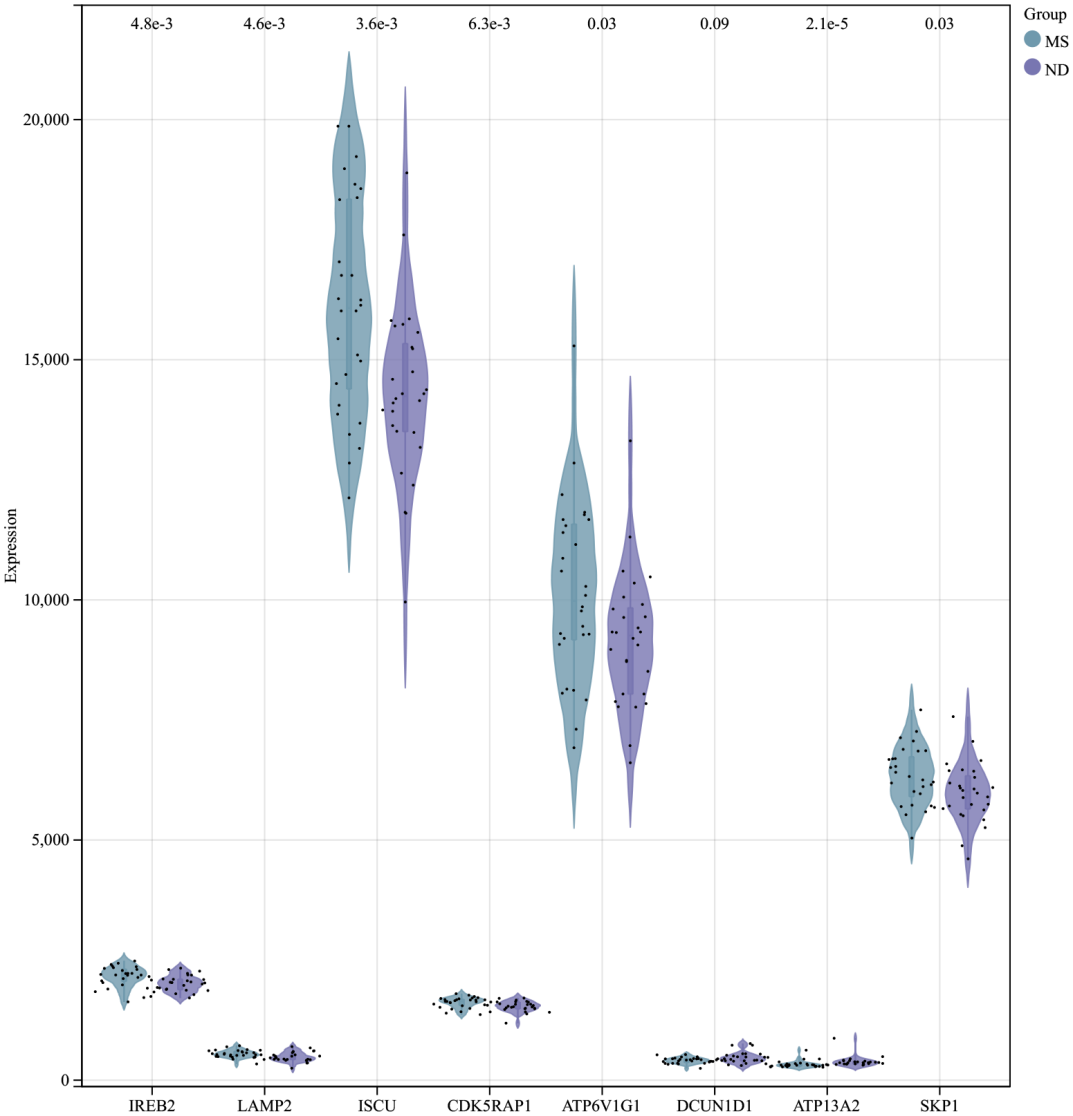
Expression of hub genes in the MS and control groups of the MS experimental samples.

Using the selection criteria “p_adj < 0.05, abs(logFC) > 0.585”, we obtained 2967 differentially expressed genes from the GSE78244 database. Details of these differentially expressed genes are provided in the **Supplementary Table S1**). The volcano plot (**Figure 2A**) and heatmap (**Figure 2B**) display the top 50 differentially expressed genes. After removing outlier samples and filtering genes, a dataset containing 25298 genes and 56 samples was used to construct a weighted gene co-expression network. When the soft threshold power was set to 4, the scale independence reached 0.89 (**Figure 3A**), and the average connectivity was 439.86 (**Figure 3B**). Sample clustering (**Figure 3C**) and gene clustering (**Figure 3D**) were used to identify samples and genes with similar expression patterns, aiding in understanding the relationships between genes and the differential expression patterns among different samples. Hub genes obtained from limma analysis, iron metabolism-related genes obtained from WGCNA, and iron metabolism genes were analyzed. The Venn diagram results are provided in the **Supplementary Table S2**), revealing 8 common genes (**Figure 4A**): IREB2, LAMP2, ISCU, CDK5RAP1, ATP6V1G1, DCUN1D1, ATP13A2, and SKP1. Except for LAMP2 and DCUN1D1, the other six genes showed differential expression between the MS and control groups, with ATP13A2 being higher in the control group, while the rest were higher in the MS group. Violin plots are depicted in **Figure 5**.

### 3.2 Biological processes and pathways enriched for the hub genes

**Figure 6:**
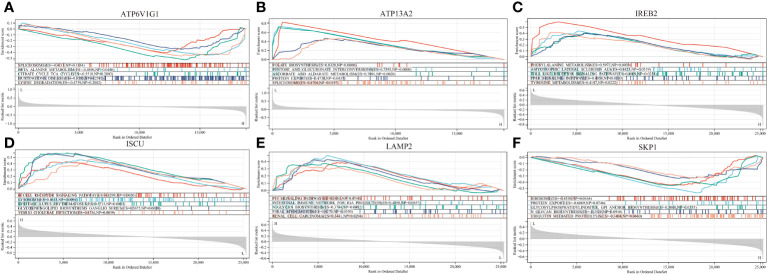
GSEA revealed the enriched pathways of the hub genes. **(A)** ATP6V1G1. **(B)** ATP13A2. **(C)** IREB2. **(D)** ISCU. **(E)** LAMP2. **(F)** SKP1.

To understand the potential biological roles of these genes, enrichment analysis was conducted. The GO analysis revealed that 6 out of the 8 genes were involved in related biological processes, including cellular iron ion homeostasis, iron ion homeostasis, and iron ion transport, all these genes were related to iron metabolism (**Figure 4B**). Subsequently, GSEA analysis of these genes showed associations with several neurodegenerative diseases (In Huntington’s disease, Amyotrophic lateral sclerosis preceded by e.g.) and pathways related to MS (GLYCOSPHINGOLIPID_BIOSYNTHESIS_GANGLIO_SERIES, B_CELL_RECEPTOR_SIGNALING_PATHWAY, Toll-like Receptor Signaling Pathway), as well as other pathways such as GLYCOSYLPHOSPHATIDYLINOSITOL_GPI_ANCHOR_BIOSYNTHESIS, TYROSINE_METABOLISM, and LYSOSOME (**Figure 6**).

### 3.3 Construction of diagnostic model and brain validation

**Figure 7:**
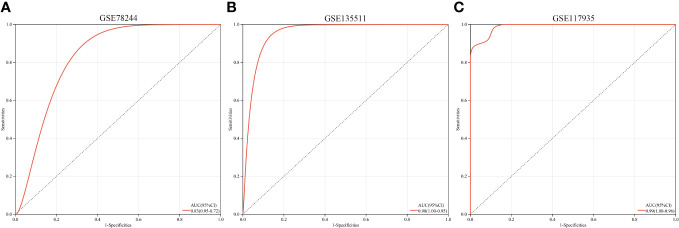
ROC curves and corresponding AUC values for the three expression cohorts. **(A)** samples from GSE78244. **(B)** Blood samples from GSE135511. **(C)** Blood samples from GSE117935.

A multi-gene prediction model derived from stepwise logistic regression analysis was developed and found to demonstrate strong diagnostic performance, as evidenced by an AUC of 0.83 (**Figure 7A**). Furthermore, the model was subsequently validated in blood samples, showing high AUC values in GSE135511 and GSE117935, with values of 0.98 and 0.99, respectively (**Figures 7B, C**). These encouraging outcomes in brain samples indicate the potential clinical utility of the model for diagnosing MS patients.

#### 3.3.1 Results of MR

**Figure 8:**
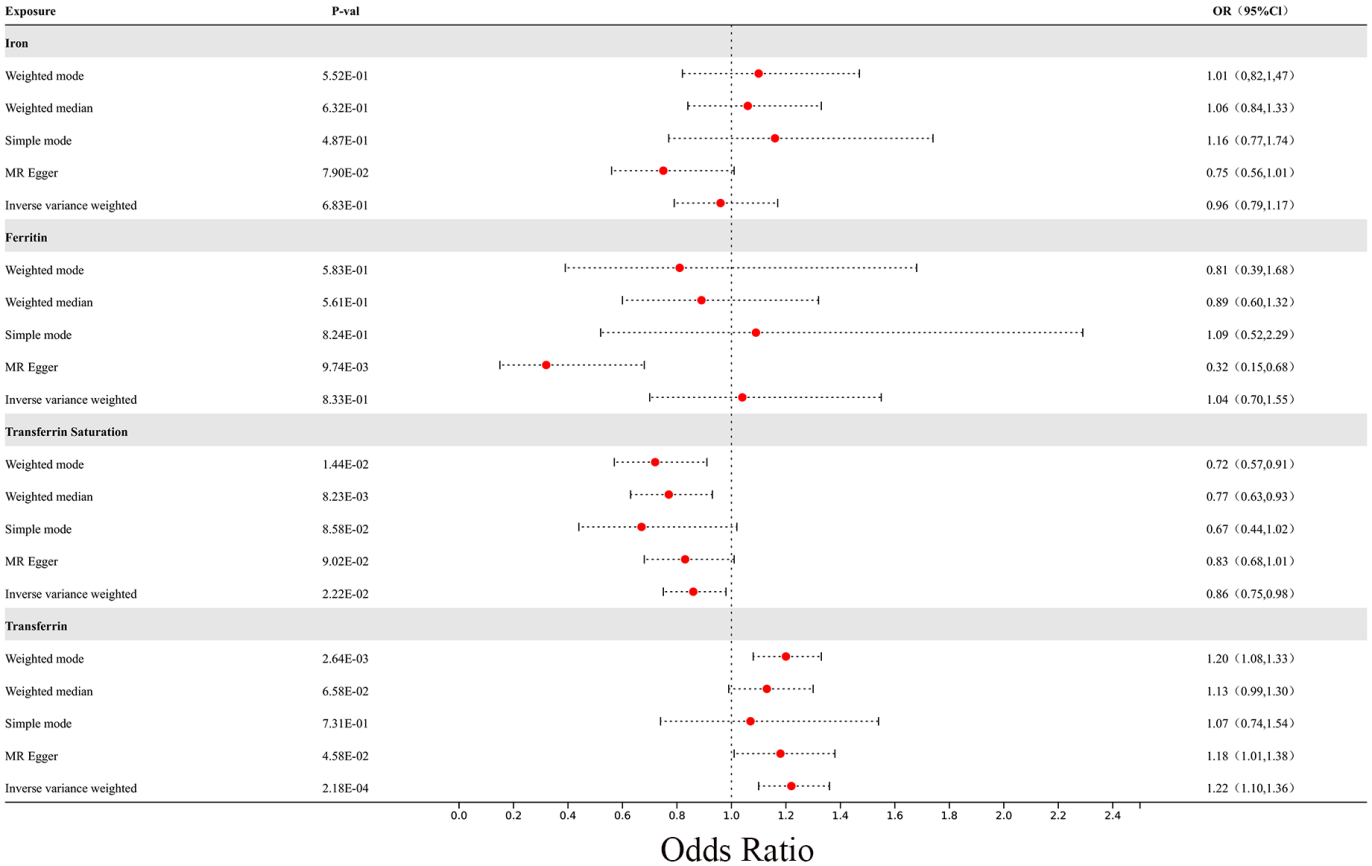
The forest plot of Mendelian randomization results for 4 different iron metabolism markers and MS.

In the MR analysis, we observed potential causal relationships between transferrin saturation, serum transferrin receptor, and MS. The IVW method results of the MR analysis are as follows: transferrin (p= 2.18E-04; OR 95%CI= 1.22 (1.10, 1.36)), transferrin saturation (p= 2.22E-02; OR 95%CI= 0.86 (0.75, 0.98)), iron (p= 6.83E-01; OR 95%CI= 0.96 (0.79, 1.17)), and serum ferritin (p= 8.33E-01; OR 95%CI= 1.04 (0.70, 1.55)) (**Figure 8**). Further MR methods are detailed in the **Supplementary Table S3**). To mitigate the potential influence of MS on iron levels, we conducted a reverse MR study. The IVW results of the reverse MR analysis are as follows: MS and iron (p= 7.88E-01; OR 95%CI= 0.99 (0.96, 1.03)), MS and Ferritin (p= 4.32E-01; OR 95%CI= 1.01 (0.98, 1.04)), MS and transferrin saturation (p= 9.04E-01; OR 95%CI= 1.00 (0.96, 1.04)), MS and serum transferrin (p= 4.47E-01; OR 95%CI= 0.99 (0.95, 1.02)). The detailed results of the reverse MR analysis are available in the **Supplementary Table S4**). Our findings suggest that the risk of MS is influenced by iron metabolism.

**Figure 9:**
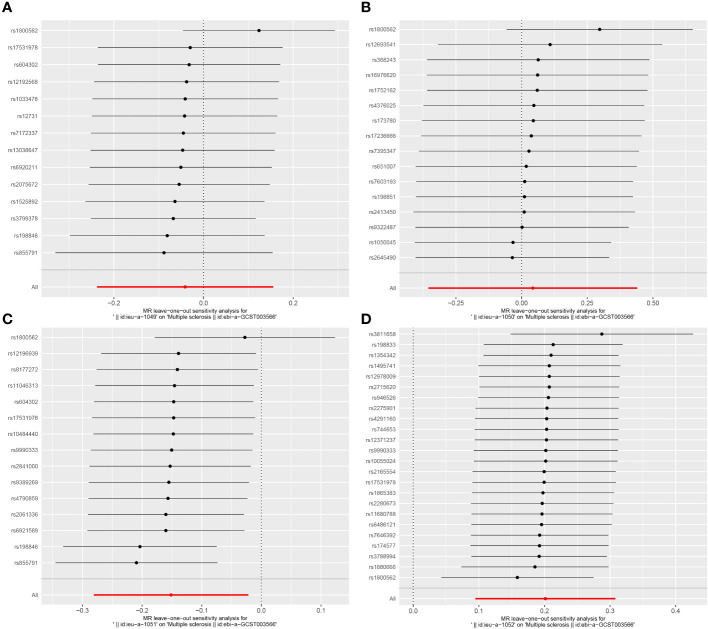
Leave-one-out results plot for **(A)** Iron and multiple sclerosis, **(B)** Ferritin and multiple sclerosis, **(C)** Transferrin Saturation and multiple sclerosis, **(D)** Transferrin and MS.

**Figure 10:**
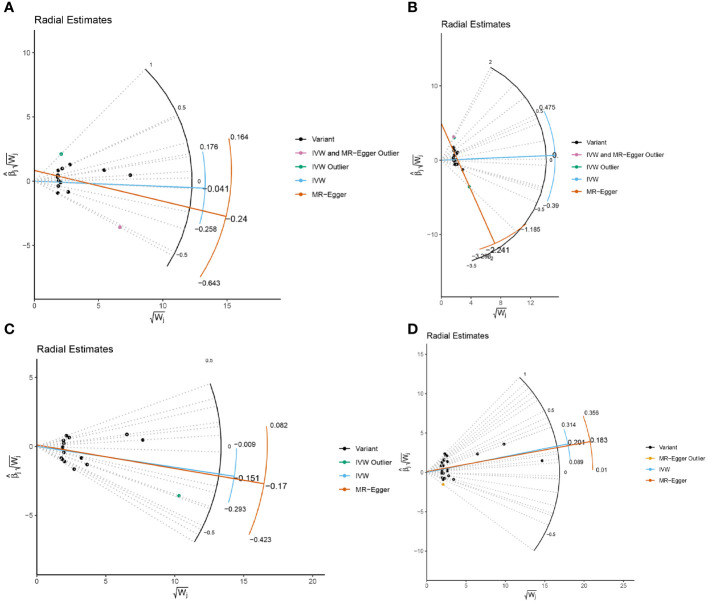
Radial MR results plot for **(A)** Iron and multiple sclerosis, **(B)** Ferritin and multiple sclerosis, **(C)** Transferrin Saturation and multiple sclerosis, **(D)** Transferrin and MS.

In the sensitivity analysis, we utilized the Cochran Q method, MR-Egger, and MR-PRESSO to assess heterogeneity and pleiotropy. All the results were greater than 0.05, indicating no evidence of heterogeneity and pleiotropy in the SNPs, as shown in **Table 1**. Additionally, we conducted a leave-one-out analysis, which also demonstrated the stability of our results (**Figure 9**). Finally, we used Radial MR to test for outliers, and the results showed that there was no interference from outliers in transferrin saturation and serum transferrin receptor, as indicated in the test results (**Figure 10**). The Radial MR results are available in the **Supplementary Table S4**).

**Table 1:** Heterogeneity and horizontal pleiotropy analysis between iron markers and MS.

Exposure	Heterogeneity				Horizontal pleiotropy			MR-PRESSO P value
	method	Q	Q_df	Q_pval	egger_intercept	se	pval	
Iron	MR Egger	3.84	6	0.70	0.03	0.05	0.61	
	IVW	4.13	7	0.76				0.8
Ferritin	MR Egger	5.34	6	0.50	0.04	0.07	0.56	
	IVW	5.72	7	0.57				0.07
Transferrin Saturation	MR Egger	9.23	7	0.24	0.00	0.05	0.93	
	IVW	9.24	8	0.32				0.26
Transferrin	MR Egger	15.27	14	0.36	0.04	0.03	0.20	
	IVW	17.29	15	0.30				0.25

#### 3.3.2 Replication and meta-analysis

**Figure 11:**
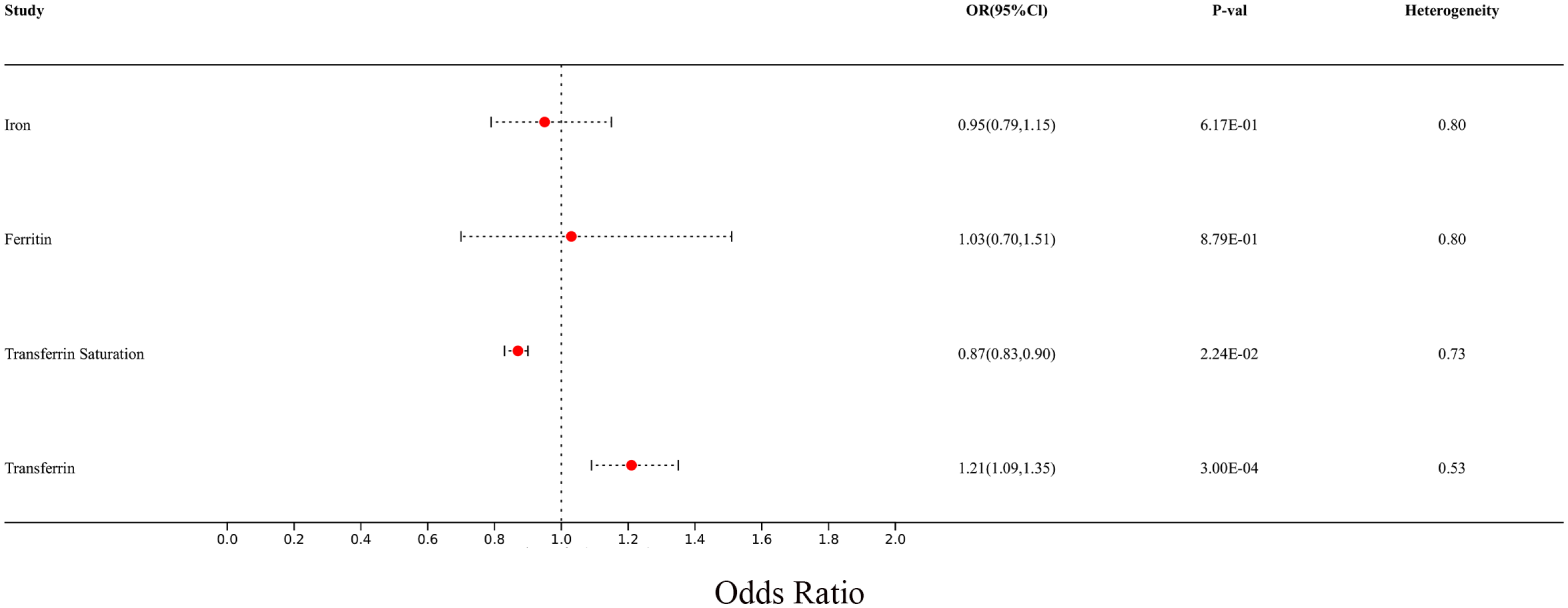
Forest plot of meta-analysis results for multiple sclerosis from two different sources.

In the meta-analysis, MS with at least two reliable MR results were integrated. The summarized results of the meta-analysis are shown in the additional file (**Supplementary Table S6**). We found that there is still a significant relationship between transferrin and transferrin saturation with MS after integrating two MR results from different data sources. The meta-analysis results using the fixed effect model are as follows: Iron (p= 6.17E-01; OR 95%CI= 0.95(0.79,1.15)), Ferritin (p=8.79E-01; OR 95%CI= 1.03(0.70,1.51)), Transferrin Saturation (p= 2.24E-02; OR 95%CI= 0.87(0.83,0.90)), Transferrin (p= 3.00E-04; OR 95%CI= 1.21(1.09,1.35)). All meta-analysis heterogeneity tests were greater than 0.05, as shown in **Figure 11**.

## 4 Discussion

In our study, we employed a comprehensive bioinformatics analysis to demonstrate the role of iron metabolism in MS. Additionally, we utilized MR methods to analyze the causal relationship between different iron statuses and MS, further substantiating the significant role of iron in MS. In our MR results, we found that transferrin levels are associated with an increased risk of MS, while transferrin saturation is associated with a decreased risk of MS. Transferrin is a potent chelator that tightly but reversibly binds to iron (34). Transferrin saturation refers to the proportion of transferrin bound to iron in the blood (35). An increase in transferrin concentration is a sign of iron deficiency. When iron levels are low, transferrin concentration typically increases to regulate iron homeostasis in the blood. An increase in transferrin saturation indicates an increase in iron levels in the blood. Therefore, our results suggest that iron deficiency may be an important contributing factor to MS. The role of transferrin is in the transport and metabolism of iron. Iron binds to transferrin and crosses the blood-brain barrier (BBB) or enters the cerebrospinal fluid, facilitating the transport of iron from the blood to the brain (36). Transferrin plays a role in iron transport within oligodendrocytes. Oligodendrocytes are the main type of iron-containing cells in the brain, and transferrin helps these cells acquire the necessary iron from the surrounding environment (37). Additionally, transferrin may be involved in regulating the utilization and storage of iron within oligodendrocytes. Once transferrin transports iron into oligodendrocytes, the iron can be utilized for cellular metabolic activities or stored in iron-binding proteins (38). This intrinsic regulation helps maintain appropriate iron levels within oligodendrocytes (39). Iron is an essential factor for myelin sheath formation, and oligodendrocytes are the main producers of myelin sheaths. Iron directly participates in cholesterol and lipid biosynthesis, which are necessary for myelin sheath formation (40).

However, an excess of iron may also contribute to the progression of MS. Numerous studies have indicated the presence of abnormal iron deposition in the brains of MS patients, which is not only localized to specific brain regions but also correlated with clinical features (41, 42). In a study involving 30 MS patients and 15 healthy controls, the researchers analyzed of quantitative susceptibility maps reconstructed from 3T magnetic resonance brain images. The results revealed regional differences in iron concentrations in MS lesions, normal-appearing white matter, thalamus, and basal ganglia, despite similar brain iron loads in MS patients and controls after adjusting for brain volume. These regional iron loads were also associated with age, disease duration, Expanded Disability Status Scale scores, Timed 25-Foot Walk test results, and disease-modifying therapy duration (8). Similar results have been reported in other studies (9, 43).

Iron can influence the progression of MS through various mechanisms. Iron metabolism dysregulation has been shown to be associated with pathogenic T lymphocytes. Studies have demonstrated that iron can promote the differentiation and function of pathogenic T lymphocytes. Specifically, the iron-dependent production of granulocyte-macrophage colony-stimulating factor is associated with the binding of iron in the form of Fe^2+^ and the stabilization of the RNA-binding protein Poly(rC) Binding Protein 1 (44). This process further promotes the transition of T cells to a more inflammatory and pathogenic phenotype, potentially exacerbating the development of autoimmune diseases (45).

These findings highlight the complex interplay between iron and MS, shedding light on the potential mechanisms through which iron influences the pathogenesis and progression of the disease. Further research in this area is crucial for a deeper understanding of the role of iron in MS and the development of targeted therapeutic interventions. Furthermore, iron can influence the function of T cells by regulating their metabolism (46). Iron has been shown to promote the glycolysis and oxidative phosphorylation (OXPHOS) of T cells, leading to the acquisition of more pathogenic metabolic characteristics (47, 48). These metabolic features may further promote the transition of T cells to a more inflammatory and pathogenic phenotype. Excessive accumulation of iron may also lead to iron-mediated lipid peroxidation, triggering ferroptosis (49, 50). This iron-mediated cell death mechanism has been implicated in the pathogenesis of MS (51, 52). Ferroptosis is a novel iron-related form of non-apoptotic cell death, distinct from other known cell death mechanisms such as apoptosis (53). It is initiated by lipid peroxidation, generating harmful lipid peroxides within the cell, ultimately leading to cell death (54). This process may be related to the loss of oligodendrocytes (OL) in the central nervous system (55). Oligodendrocytes have high levels of iron and are highly sensitive to oxidative stress due to their low levels of the antioxidant enzyme glutathione and high iron content (56). Therefore, the content and metabolism of iron in oligodendrocytes may have a significant impact on the development and progression of MS.

The strengths of this study include the use of multiple methods, including bioinformatics analysis and MR methods, to comprehensively investigate the relationship between iron and MS from different perspectives, making the research results more comprehensive and reliable. Additionally, the study utilized publicly available databases and a large amount of gene expression data, providing a solid foundation for the research. Finally, the study employed rigorous statistical analysis methods, including stepwise regression analysis, logistic regression models, and MR methods, to ensure the reliability and accuracy of the research results. However, there are also some limitations. Due to the lack of GWAS data on iron-related hub genes, we were unable to validate the relationship between the genes obtained from comprehensive bioinformatics analysis and MS using MR methods. In addition, the data used in this study were derived from European population databases, and the results may not be generalizable to all populations.

## 5 Conclusion

This study provides an in-depth exploration of the relationship between iron and MS using multiple methods, offering important insights for further research on the role of iron in the pathogenesis of MS. These findings are expected to provide new perspectives and directions for the prevention and treatment of related diseases, offering important theoretical support for clinical practice and treatment strategies.

## Data availability statement

The original contributions presented in the study are included in the article/**Supplementary Material**. Further inquiries can be directed to the corresponding author.

## Author contributions

CT: Conceptualization, Software, Writing – original draft. JY: Data curation, Methodology, Writing – original draft. CZ: Data curation, Writing – original draft. YD: Formal analysis, Writing – original draft. SY: Visualization, Writing – original draft. BX: Visualization, Writing – original draft. DH: Funding acquisition, Resources, Writing – review & editing.
